# 异基因造血干细胞移植后腺病毒感染26例诊治分析

**DOI:** 10.3760/cma.j.issn.0253-2727.2023.04.007

**Published:** 2023-04

**Authors:** 斐 周, 苏宁 陈, 德沛 吴, 雪峰 何

**Affiliations:** 苏州大学附属第一医院血液科，国家血液系统疾病临床医学研究中心，江苏省血液研究所，苏州 215006 Department of Heamatology, the First Affiliated Hospital of Soochow University, National Clinical Research Center for Hematology, Jiangsu Institute of Hematology, Soochow 215006, China

**Keywords:** 异基因造血干细胞移植, 腺病毒, Allogeneic hematopoietic stem cell transplantation, Adenovirus infection

## Abstract

**目的:**

分析异基因造血干细胞移植后腺病毒感染患者的临床特征及预后。

**方法:**

收集2018–2022年苏州大学附属第一医院移植后病房收治的26例异基因造血干细胞移植后腺病毒感染患者，分析患者基本资料、临床特征、治疗和随访情况。

**结果:**

26例患者中位年龄30（22，44）岁。22例（84.62％）接受亲缘单倍体造血干细胞移植，3例（11.54％）接受无关移植，1例（3.85％）接受脐血干细胞移植。25例（96.15％）预处理方案中包含ATG。腺病毒感染发生的中位时间为移植后95（44，152）d。中位外周血淋巴细胞计数为0.30（0.11，0.69）×10^9^/L。12例（46.15％）合并急性移植物抗宿主病。24例（92.31％）在诊断时仍接受抗排异治疗。16例（61.54％）患者除腺病毒感染外，合并其他感染。8例（30.77％）为无症状感染，18例（69.23％）为腺病毒病：肺炎（38.89％）、胃肠炎（38.89％）、脑炎（33.33％）、肝炎（5.56％）、泌尿道炎症（5.56％）。年龄>30岁是发生腺病毒病的危险因素（*P*＝0.03）。18例（69.23％）进行免疫抑制剂减撤治疗，所有患者均使用至少一种抗病毒药物，其他治疗包括大剂量丙种球蛋白及供者淋巴细胞输注。10例（38.46％）腺病毒感染好转，16例（61.54％）进展或无效。中位随访时间为30（7，237）d，22例患者死亡。全因死亡率为（88.5±7.1）％，归因死亡率为45.5％，无症状感染者与腺病毒病患者100 d生存率差异无统计学意义（37.5％对22.2％, *HR*＝1.83, 95％ *CI* 0.66～5.04, *P*＝0.24）。

**结论:**

年龄>30岁是发生腺病毒病的危险因素，异基因造血干细胞移植后腺病毒感染患者死亡率较高。

## Abstract (en)

**Objective:**

To analyze the clinical characteristics and prognosis of adenovirus infection after allogeneic hematopoietic stem cell transplantation.

**Methods:**

A total of 26 patients with adenovirus infection admitted to the posttransplant ward of the First Affiliated Hospital of Soochow University from 2018 to 2022 were enrolled. Their data on baseline and clinical characteristics, treatment, and follow-up were analyzed.

**Results:**

The median patient age was 30（22，44）years. Twenty-two patients received related haploid stem cell transplantation, three received unrelated stem cell transplantation, and one received umbilical cord stem cell transplantation. Antithymocyte globulin was included in the conditioning regimen in 25 patients. The median time of adenovirus infection was +95（+44，+152）days. The median peripheral blood lymphocyte count was 0.30（0.11，0.69）× 10^9^/L. Twelve patients had acute graft-versus-host disease. Twenty-four patients received antirejection therapies at diagnosis. Sixteen cases had combined infection with other pathogens with adenovirus infection. Eight cases were diagnosed as asymptomatic infection, and 18 were diagnosed as adenovirus disease, including pneumonia（38.89％）, gastrointestinal disease（38.89％）, encephalitis（33.33％）, hepatitis（5.56％）, and urinary tract inflammation（5.56％）. The age of >30 years was a risk factor for adenovirus disease（*P*＝0.03）. Eighteen patients received tapering of immunosuppression, and all 26 patients received at least one antiviral drug. Other treatments included high-dose gamma globulin and donor lymphocyte infusion. Adenovirus infection improved in 10 cases and progressed in 16 cases. The median follow-up time was 30（7, 237）days. Twenty-two patients died. The all-cause mortality rate was（88.5±7.1）％, and the attributable mortality rate was 45.5％. There was no significant difference in the 100 d survival rate between asymptomatic infected patients and patients diagnosed with adenovirus disease（37.5％*vs* 22.2％, *HR*＝1.83, 95％ *CI* 0.66–5.04, *P*＝0.24）.

**Conclusion:**

The age of >30 years was a risk factor for adenovirus disease. Mortality was high in patients with adenovirus infection after allogeneic hematopoietic stem cell transplantation.

人腺病毒是一组DNA病毒，分A～G 7型，包括80余种血清型[1]。免疫力正常的个体及免疫抑制个体均可发生腺病毒感染。腺病毒感染是异基因造血干细胞移植（allo-HSCT）后相对常见的感染并发症，发生率为5％～21％[2]–[3]，死亡率达50％[4]。根据欧洲白血病感染会议（ECIL-4）指南[5]，腺病毒感染的危险因素包括：脐血干细胞移植、接受去T细胞或无关供者移植的儿童患者、阿仑单抗预处理或单倍体造血干细胞移植的成年患者、严重淋巴细胞缺乏（<0.2×10^9^/L）、3～4级急性移植物抗宿主病（GVHD）等。腺病毒在免疫抑制个体中可导致多种临床综合征，从无症状病毒血症，到有局部表现的急性呼吸道疾病、胃肠炎、结膜炎或尿路感染等，甚至到播散性疾病，严重者可导致单个或多个脏器致命性衰竭[6]。本文我们回顾性总结了26例allo-HSCT后腺病毒感染病例的临床特征及预后，报道如下。

## 病例与方法

一、病例资料

本研究为回顾性研究，以2018年6月1日至2022年5月31日在苏州大学附属第一医院移植后发生腺病毒感染的26例患者为研究对象。收集患者的临床资料，包括诊断时的年龄、性别、血液病诊断、预处理方案、移植类型、是否合并急性GVHD、是否二次移植、诊断时外周血淋巴细胞计数、免疫抑制剂使用情况、是否合并感染、治疗情况、转归等。GVHD的诊断及分级参照MAGIC标准[7]。

二、腺病毒感染诊断

目前无公认的腺病毒感染或腺病毒病的统一定义。本研究参照La Rosa等[8]研究中的定义。本研究中所有患者腺病毒存在的证据均通过病原体高通量检测手段对外周血、粪便、脑脊液、肺泡灌洗液或尿液检测得到，结合相应器官临床表现及体征，排除其他可能引起相应症状体征的病因，从而进行相应的诊断。

三、随访

随访截止日期为2023年3月31日。中位随访30（7，237）d。主要观察指标为患者腺病毒感染的转归。全因死亡率定义为随访期内任何原因导致死亡的人数占总人数的比例。归因死亡率定义为随访期内由于腺病毒感染导致死亡的人数占任何原因导致死亡人数的比例。100 d生存率定义为从腺病毒感染确诊日期起100 d内患者存活率。

四、统计学处理

使用R 4.1.2进行统计分析。利用Kaplan-Meier方法绘制生存曲线，Log-rank检验比较两组100 d生存率的差异。分类变量和连续变量（数据不符合正态分布）分别以例数（构成比）和中位数（*IQR*）描述，采用Fisher精确概率法和Mann-Whitney *U*检验进行差异性比较。*P*<0.05为差异有统计学意义。

## 结果

一、病例特征

本中心近4年行allo-HSCT共3 343例，移植后病房共收治了26例腺病毒感染患者，估算腺病毒感染发生率为0.78％。26例allo-HSCT后发生腺病毒感染的患者中，男18例（69.23％），女8例（30.77％），中位年龄30（22，44）岁。基础疾病：急性髓系白血病10例（38.46％），急性淋巴细胞白血病7例（26.92％），急性混合表型白血病1例（3.85％），重型再生障碍性贫血3例（11.54％），骨髓增生异常综合征2例（7.69％），慢性髓性白血病（急淋变）、Burkitt淋巴瘤、慢性粒-单核细胞白血病各1例（3.85％）。24例（92.31％）患者为首次移植，2例（7.69％）患者为二次移植。其中22例（84.62％）患者接受了亲缘来源的单倍体造血干细胞移植，2例（7.69％）接受了无关全相合造血干细胞移植，1例（3.85％）接受9/10相合无关造血干细胞移植，1例（3.85％）接受双份脐血干细胞移植。预处理方案：23例（88.46％）患者为改良白消安/环磷酰胺（Bu/Cy）+抗胸腺细胞球蛋白（ATG），1例（3.85％）为全身放射治疗（TBI）+CY+ATG，1例（3.85％）为FBC（氟达拉滨、白消安、环磷酰胺）+ATG，1例（3.85％）为Flu+Bu/Cy+阿糖胞苷（Ara-C）+G-CSF。25例（96.15％）患者预处理方案中均包含ATG。

腺病毒感染发生距离移植的中位时间为95（44，152）d，其中13例（50.00％）患者腺病毒感染发生在移植后100 d内，13例（50.00％）发生时间≥移植后100 d。26例患者中，24例（92.31％）感染1种类型腺病毒，其中A型2例（7.69％）、B型4例（15.38％）、C型15例（57.69％）、D型2例（7.69％）、F型1例（3.85％）；1例（3.85％）同时感染C、D两种类型腺病毒，1例（3.85％）同时感染B、D、E三种类型腺病毒。26例患者发生腺病毒感染时中位外周血淋巴细胞计数为0.30（0.11，0.69）×10^9^/L，其中9例（34.62％）<0.2×10^9^/L，17例（65.38％）≥0.2×10^9^/L。12例（46.15％）患者发生腺病毒感染时合并急性GVHD，其中10例（38.46％）为4级aGVHD；4例（15.38％）患者发生腺病毒感染时合并慢性GVHD。24例（92.31％）患者在诊断腺病毒感染时仍在使用抗排异治疗，其中23例（88.46％）使用糖皮质激素，21例（80.77％）使用钙调磷酸酶抑制剂（环孢素A或他克莫司），15例（57.69％）使用芦可替尼，9例（34.62％）使用CD25单抗，4例（15.38％）使用TNF单抗，3例（11.54％）使用吗替麦考酚酯，2例（7.69％）使用维得利珠单抗，1例（3.85％）使用环磷酰胺，1例（3.85％）使用间充质干细胞，1例（3.85％）患者使用IL-6单抗。1例（3.85％）患者仅使用糖皮质激素一种抗排异药物，8例（30.77％）使用2种抗排异药物，6例（23.08％）使用3种抗排异药物，3例（11.54％）使用4种（15.38％）抗排异药物，6例（23.08％）使用5种抗排异药物。16例（61.54％）患者除腺病毒感染外，合并其他感染：8例（30.77％）、4例（15.38％）、3例（11.54％）、1例（3.85％）患者分别感染1、2、3、4种病原体。10例（38.46％）合并巨细胞病毒激活，其中4例（15.38％）发展为巨细胞病毒病。6例（19.23％）患者合并EB病毒激活，其中4例（15.38％）EB病毒病。4例（15.38％）患者同时存在巨细胞病毒和EB病毒激活。共8例（30.77％）患者合并除巨细胞病毒、EB病毒以外的其他感染，包括真菌感染5例（19.23％），细菌5例（19.23％），耶氏肺孢子菌1例（3.85％）。

26例腺病毒感染患者中，8例（30.77％）为无症状感染者，18例（69.23％）诊断为腺病毒病。腺病毒病患者中10例（55.56％）为确诊病例（5例肺炎、4例脑炎、1例泌尿系感染），1例确诊腺病毒脑炎患者临床诊断合并肝脏、胃肠道、肺脏受累；8例（44.44％）为临床诊断病例（6例胃肠炎、1例脑炎、1例肺炎合并脑炎）。无症状腺病毒感染及腺病毒病患者的临床特征见表1，两组在性别、移植类型、发生时间、外周血淋巴细胞计数、是否合并其他感染、是否发生GVHD、是否使用芦可替尼、免疫抑制剂种类等方面差异均无统计学意义（均*P*>0.05）；腺病毒病组中年龄>30岁患者比例明显高于无症状腺病毒感染组（Fisher，*P*＝0.030）。

**表1:** 无症状腺病毒感染及腺病毒病患者的临床特征比较［例数（％）］

临床特征	无症状腺病毒感染（8例）	腺病毒病（18例）	*P*值
性别			0.667
男	5（62.50）	13（72.22）	
女	3（37.50）	5（27.78）	
年龄			0.030
≤30岁	7（87.50）	6（33.33）	
>30岁	1（12.50）	12（66.67）	
HLA相合			1.000
全相合	0（0.00）	2（11.11）	
不全相合	8（100.00）	16（88.89）	
腺病毒感染距移植时间			0.673
<100 d	5（62.50）	8（44.44）	
≥100 d	3（37.50）	10（55.56）	
外周血淋巴细胞计数			0.209
>0.4×10^9^/L	3（37.50）	4（22.22）	
（0.2~0.4）×10^9^/L	1（12.50）	9（50.00）	
<0.2×10^9^/L	4（50.00）	5（27.78）	
急性GVHD			0.573
无	4（50.00）	6（33.33）	
1~2级	0（0.00）	1（5.56）	
3~4级	4（50.00）	7（38.89）	
慢性GVHD	0（0.00）	4（22.22）	
GVHD治疗药物种类			0.785
0	1（12.50）	1（5.56）	
1	0（0.00）	1（5.56）	
2	3（37.50）	5（27.78）	
≥3	4（50.00）	11（61.11）	
使用芦可替尼			0.218
是	3（37.50）	12（66.67）	
否	5（62.50）	6（33.33）	
合并感染			0.664
是	4（50.00）	12（66.67）	
CMV激活	3（37.50）	7（38.89）	1.000
EBV激活	1（12.50）	5（27.28）	0.628
否	4（50.00）	6（33.33）	

**注** CMV：巨细胞病毒；EBV：EB病毒

二、治疗

24例（92.31％）患者在诊断腺病毒感染时仍在使用免疫抑制剂，其中18例（75.00％）患者在诊断后进行免疫抑制剂减撤治疗，6例（25.00％）患者因处于移植后早期（2例，8.33％）或合并3～4级GVHD（4例，16.67％）而未进行免疫抑制剂减撤。13例（50.00％）患者接受了西多福韦治疗，其中12例为腺病毒病患者，1例为无症状腺病毒感染患者。西多福韦使用方法为每次5 mg/kg，每周1次×2周，后每2周1次，7例（26.92％）患者接受了至少2剂西多福韦治疗，6例（23.08％）患者仅接受了1剂西多福韦治疗。患者所接受的其他抗病毒治疗包括：利巴韦林13例（50.00％）、膦甲酸钠7例（26.92％）、更昔洛韦4例（15.38％）、阿昔洛韦4例（15.38％）、大剂量丙种球蛋白冲击8例（30.77％）、小剂量供者淋巴细胞输注（DLI）1例（3.85％）。所有26例患者除减撤免疫抑制剂外，均使用至少1种抗病毒药物。13例（50.00％）、8例（30.77％）、1例（3.85％）、4例（15.38％）患者分别接受1、2、3、4种抗病毒治疗。

三、转归及预后

26例患者中，10例（38.46％）患者临床症状好转，16例（61.54％）患者临床症状进展或无变化。18例腺病毒病患者中，6例临床症状好转，12例进展或无变化。8例无症状腺病毒感染患者中，4例好转，4例进展或无变化。

13例接受西多福韦治疗患者中4例（30.77％）病毒转阴，且临床症状好转，中位起效时间为2（1，3）周；其中1例合并使用DLI，病毒未转阴，且临床症状无好转，最终死于腺病毒病（多脏器受累）。未使用西多福韦治疗的13例患者中 6例（46.15％）好转。是否使用西多福韦疗效差异无统计学意义（*P*＝0.688）。

本研究中，22例患者死亡，全因死亡率为（88.5±7.1）％。其中10例死于腺病毒感染，归因死亡率为45.5％；5例（22.72％）死于其他病原体感染，2例（9.09％）死于血液病复发，1例（4.55％）死于重度移植物抗宿主病，1例（4.55％）死亡多脏器功能衰竭，1例（4.55％）死于移植相关血栓性微血管病，2例（9.09％）死亡原因不明。患者自诊断腺病毒感染至死亡中位生存时间为25（0，73）d。8例无症状感染患者中，5例（62.50％）死亡（无一例死于腺病毒感染），3例（37.50％）存活。18例腺病毒病患者中，17例（94.44％）死亡（10例死于腺病毒感染），仅1例（5.56％）存活。无症状腺病毒感染及腺病毒病两组患者100 d生存率差异无统计学意义，分别为37.5％和22.2％（*HR*＝1.83，95％*CI* 0.66～5.04，*P*＝0.24）。

## 讨论

本研究中腺病毒感染发生率0.78％，明显低于既往文献所述的5％～21％[2]–[3]，可能因为本中心常规不监测腺病毒，只在发热或有相应临床症状患者中进行检测，故腺病毒感染，尤其是无症状感染发生率被大大低估。既往研究表明，腺病毒病在腺病毒感染患者中占20％～89％[8]–[11]，而本组病例中占64.29％。鉴于本中心不常规监测腺病毒，因此无法早期发现腺病毒感染并进行相应干预，因此腺病毒病比例偏高。

本研究中50％患者腺病毒感染发生在移植后100 d内，所有患者在感染发生时外周血淋巴细胞计数均低于正常值下限，34.62％的患者<0.2×10^9^/L。既往研究表明，在移植后100 d内，因缺乏功能性CD4^+^/CD8^+^ T淋巴细胞，更容易发生腺病毒的激活[12]。其他的危险因素包括：低龄、3～4级急性GVHD、脐血干细胞移植、不匹配或不相关的供者移植、体内或体外去T细胞等[13]。本研究中所有患者均非同胞全相合造血干细胞移植，84.62％为亲缘单倍体造血干细胞移植，且除1例脐血干细胞移植外，96.15％患者预处理方案中均包含ATG，42.31％的患者合并3～4级急性GVHD，92.31％的患者在诊断时仍在接受抗GVHD治疗，与上述危险因素相符。

既往研究表明，严重的淋巴细胞减少（<0.2×10^9^/L）、持续的免疫抑制、外周血腺病毒PCR阳性、3～4级GVHD和多部位分离出病毒是腺病毒感染发展为腺病毒病的危险因素[8],[14]。而在本研究中，无症状腺病毒感染患者与腺病毒病患者相比，仅在发病年龄方面差异有统计学意义，其他临床特点差异无统计学意义。由于本研究样本量偏小，需纳入更多病例进一步验证。

目前，对于腺病毒感染的监测、抢先治疗时机及治疗原则并无统一定论。根据ECIL指南[15]，建议根据外周血CD3^+^ T细胞计数、是否使用免疫抑制治疗、是否存在外周血腺病毒阳性、是否正在接受抗病毒治疗，每周监测外周血腺病毒1～2次（qPCR），并且根据是否高危患者、是否存在粪便腺病毒阳性高拷贝数，评估腺病毒特异性T淋巴细胞。但是，腺病毒感染高危人群需要进一步研究确定[16]。更昔洛韦的抢先治疗已被证明可降低巨细胞病毒病的发生，但腺病毒血症的抢先治疗意义尚不明确。ECIL指南推荐的治疗按推荐级别从高到低分别为：免疫抑制剂减量、西多福韦（1 mg/kg，1周3次，联合丙磺舒和充分水化）、利巴韦林（针对腺病毒C型）、更昔洛韦或膦甲酸钠[17]，西多福韦仅用于临床研究或同情使用。但是上述所有药物在腺病毒感染中的疗效仍需更多大型研究证实。本研究中，使用西多福韦治疗组与未使用西多福韦治疗组有效率差异无统计学意义，可能原因是本研究纳入样本量过小。考虑到免疫功能在抗病毒治疗中的重要性，细胞过继治疗受到越来越多的关注。一项对38例腺病毒感染患者使用第三方五价细胞毒性T淋巴细胞（CTL）治疗的研究表明，其总应答率为92％，CTL在循环中持续12周，可有助于降低腺病毒感染死亡率，并通过减少抗病毒药物的使用而减少相关不良反应[18]。其他研究亦表明病毒特异性CTL治疗可降低腺病毒感染死亡率，同时不显著增加GVHD发生率[19]–[20]，因此细胞过继治疗在抗腺病毒治疗中具有一定前景。本研究中，75％在使用免疫抑制剂的患者接受了免疫抑制剂减撤治疗，所有患者均接受了至少1种抗病毒药物治疗，包括西多福韦、利巴韦林、膦甲酸钠、更昔洛韦、阿昔洛韦等，此外还有患者接受静脉免疫球蛋白冲击治疗及小剂量DLI。本研究中，全因死亡率为88.5％，中位生存时间25 d。22例死亡患者中，10例死于腺病毒感染，且均为腺病毒病患者。由于样本量偏少，尚不足以计算影响死亡的危险因素，未来增加病例数后可进一步分析。无症状腺病毒感染患者死亡率略低于腺病毒病患者，但差异无统计学意义，可能亦与样本量小相关。本研究总死亡率高于既往研究数据，可能因为大多数患者诊断时即有脏器受累，且发现时即处于疾病晚期。因此，对腺病毒感染高危患者外周血及粪便标本、咽拭子进行常规腺病毒监测，对腺病毒阳性患者进行抢先治疗，对于改善腺病毒感染患者的预后及长期生存可能具有重要意义。此外，对有呼吸道症状的不明病原体感染的患者，有条件可考虑行支气管镜，留取肺泡灌洗液进行腺病毒检测。

综上所述，年龄>30岁是发生腺病毒病的危险因素，异基因造血干细胞移植后腺病毒感染患者死亡率较高，无症状感染者与腺病毒病患者死亡率差异无统计学意义。以上结论有待进一步验证。
